# Persistent Topology and Metastable State in Conformational Dynamics

**DOI:** 10.1371/journal.pone.0058699

**Published:** 2013-04-02

**Authors:** Huang-Wei Chang, Sergio Bacallado, Vijay S. Pande, Gunnar E. Carlsson

**Affiliations:** 1 Institute for Computational and Mathematical Engineering, Stanford University, Stanford, California, United States of America; 2 Department of Structural Biology, Stanford University, Stanford, California, United States of America; 3 Department of Chemistry, Stanford University, Stanford, California, United States of America; 4 Department of Mathematics, Stanford University, Stanford, California, United States of America; University of Leeds, United Kingdom

## Abstract

The large amount of molecular dynamics simulation data produced by modern computational models brings big opportunities and challenges to researchers. Clustering algorithms play an important role in understanding biomolecular kinetics from the simulation data, especially under the Markov state model framework. However, the ruggedness of the free energy landscape in a biomolecular system makes common clustering algorithms very sensitive to perturbations of the data. Here, we introduce a data-exploratory tool which provides an overview of the clustering structure under different parameters. The proposed *Multi-Persistent Clustering* analysis combines insights from recent studies on the dynamics of systems with dominant metastable states with the concept of multi-dimensional persistence in computational topology. We propose to explore the clustering structure of the data based on its persistence on scale and density. The analysis provides a systematic way to discover clusters that are robust to perturbations of the data. The dominant states of the system can be chosen with confidence. For the clusters on the borderline, the user can choose to do more simulation or make a decision based on their structural characteristics. Furthermore, our multi-resolution analysis gives users information about the relative potential of the clusters and their hierarchical relationship. The effectiveness of the proposed method is illustrated in three biomolecules: alanine dipeptide, Villin headpiece, and the FiP35 WW domain.

## Introduction

Molecular Dynamics (MD) simulation is a useful tool to understand biomolecular events such as protein folding at the atomic level. Insights derived from simulations have the potential to guide laboratory experiments. The development of advanced hardware, computing architecture, and algorithms in MD simulation has given researchers the power to produce massive trajectory data. For example, the Folding@Home project, which uses distributed computing on CPUs, GPUs and game consoles, has simulated protein folding up to the 10 ms timescale [1]. Besides, the special-purpose supercomputer Anton has been used to simulate 12 structurally different proteins with folding timescales up to 0.1 ms [2], [3].

Biomolecular kinetics are frequently understood in terms of a free energy landscape characterized by basins (local minima) which represent long-lived or *metastable* states. Facing increasing volume of MD simulation data, researchers need some means to help them study the free energy landscape of a molecule system from the simulation data. Direct visualization of the trajectory is an straightforward way of analyzing MD simulations. However, as the systems become more complex, these analyses provide an oversimplified picture of the conformational kinetics; global properties or rare but important events may be overlooked. Another common way of studying the MD simulation data is to project the trajectories onto a small number of reaction coordinates, designed to capture transitions between relevant metastable states. Nevertheless, it has been shown that important features of the dynamics can be hidden in such projections (f.g. [4]).

A promising alternative for studying the free energy landscape from MD simulations is to group the sampled conformations, so that the clustering of the conformations or the partition of the configuration space reflects the basins of the free energy landscape. Then the thermodynamics or kinetics of the molecule system can be summarized in a model or a graph built upon the clusters. For example, popular strategies include building a disconnectivity graph (DG) for the basins and building a Markov state model (MSM) for the metastable states.

In a disconnectivity graph, conformations are mapped by direct minimization to their nearest local minimum several times each at a different temperature or energy [5], [6]. The mapping forms a partition of the configuration space into basins, and the topology of the basins is summarized into a tree to reveal the thermodynamic and kinetic properties of underlying potential energy surface. The disconnectivity graph approach has been applied to several systems and proven to be a useful tool for providing an overview of the basins [7]. However, computing databases of minima, which requires numerical minimization of an energy or free energy function, is usually not an easy task especially for an all-atom model (f.g. [8]), and it could be sensitive to perturbation of data.

In a Markov state model (MSM) [9], [10], a finite number of metastable states are obtained by clustering the conformations based on structural similarity and kinetics, and the kinetics between the metastable states are modeled as a reversible Markov chain. The Markov state model can be represented as a graph with edge weights reflecting the kinetics between states. Although an MSM can provide clearer kinetics information among states compared to a DG, building an MSM requires much insight about the system, which is precisely what we hope to derive from our analysis of the data. For example, most clustering algorithms take as an input the number of clusters, and that is an important question we seek to answer: how many metastable states characterize the system. A common heuristic to answer this question uses the location of the largest spectral gap in a microstate transition probability matrix (c.f. [10]). Another disadvantage of an MSM comparied to a disconnectivity graph is that it only provides information at one resolution. MSMs are multi-resolution in nature; it is possible to divide a metastable state into finer states that are still metastable to some extent; knowing the hierarchical structure of the states could provide a clearer global picture of the system.

In this paper, we proposed a data exploration method, Multi-Persistent Clustering (MPC), for solving the model selection problem of MSMs by extending the topology analysis concept used in building a disconnectivity graph. The central idea of MPC is the *multi-dimensional persistence* concept in computational topology (c.f.[11]–[13]) with commute time distance. The MPC analysis provides an overview of the clustering structure at different free energy levels, by which researchers can build an MSM with states persistent across a certain range of clustering and sampling settings. This goal seems can be achieved by using a disconnectivity graph as well, but the clustering procedure in our approach is much simpler to compute. Furthermore, the commute time distance used in our approach is based on the global structure of free energy surface and is much more robust to noise and data perturbation. Compared with conventional methods of building an MSM, the clustering procedure in MPC requires little prior knowledge about the system, and it does not have the problem of being trapped in local optimums. In summary, the proposed MPC analysis aims to combine the merits of the disconnectivity graph and the Markov state model but avoid corresponding complicated optimization and model selection problems.

In the following sections, we first reviewed the conventional methods of building a MSM from MD simulations. Then, we described the MPC analysis for data exploration and model selection of MSMs, and applied it to a simulation of the terminally-capped alanine dipeptide. The energy landscape of this small molecule can be visualized in a two dimensional space, which will help illustrate the advantages of our algorithm. We also applied the method to simulations of the Villin headpiece and the FiP35 WW domain. We will see our approach can easily produce some previously published results in an interpretable approach without much fine-tuning and solving an complicated optimization problem.

## Methods

### Clustering procedure

To use both the structural similarity of conformations and kinetic information in the simulated trajectories, an MSM is usually built by a two-step procedure [10], [14]. In the first step, the conformations observed in the simulation are clustered into *microstates* according to a structural distance function, such as the root-mean-squared distance (RMSD). The algorithms used for this structure-based clustering include the 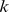-medoids, 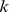-center, and hierarchical clustering methods [10], [15]. Since RMSD could be misleading when used as a surrogate for kinetics, this step needs to be done with care. Please see [16] for more discussion. The second step in the construction of an MSM uses the number of transitions observed in the simulation between different microstates to further group them into metastable states. Optimization algorithms such as PCCA [17], [18] produce a full partition of the microstates.

The first step can be viewed as a discretization step, in which we partition a continuous space of structures into a finite set of microstates. A good discretization should avoid merging conformations across barriers of the free energy landscape. As a result, there are usually hundreds to thousands of microstates. Furthermore, we would like the model resulting from the second step to contain a small number of clusters interpretable at a high level. For example, the final metastable states could correspond to the native state of the protein or an important intermediate.

The proposed MPC analysis focuses on the second step in the construction of an MSM. That is, MPC is a cluster analysis method for clustering the microstates and its input is a set of MD trajectories where each conformation is represented by a microstate index. In stead of solving a discrete optimization problem directly, our method is based on the neighborhood clustering algorithm, also known as the Vietoris-Rips complex in the computational topology literature. Given a distance function between data points and a *scale parameter*, 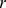, the clusters are defined as the connected components of the neighborhood graph, in which the nodes are microstates and there exists an edge between two nodes if the distance between them is no larger than the scale parameter. The output of this algorithm matches the result of a single-linkage hierarchical clustering cut at the scale 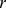.

### Commute time distance

Our clustering procedure requires a distance function in the space of microstates, which should capture the kinetic similarity between two microstates. In this paper, we will use the *commute time distance*, which we now define. For two microstates 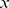 and 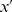, the hitting time from 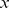 to 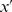 is the expected number of steps it takes a random walker which starts at 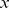 to reach 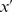 for the first time. Note that the hitting time between two microstates may not be symmetric. The *commute time* between two microstates is defined as the sum of the hitting time from 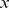 to 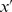 and the hitting time from 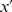 to 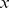. In other words, the commute time distance between 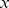 and 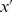 is the expected number of steps it takes a random walker starting from 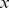 to return to 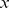 after it visits 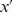. Unlike hitting time, the commute time distance is symmetric.

To estimate the commute time distance between two states, we will use a Markov model in the space of microstates. We estimate a transition probability 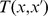 from microstate 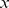 to 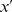 by dividing the number of transitions observed between 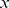 and 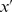 in the trajectories by the total number of transitions out of 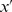. Given the transition probability matrix 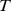, the commute time distance between two microstates is the solution of a simple linear equation [19]. There are a number of methods to assess the quality of such estimates of kinetic properties from a Markov model [16]. We expect that, even if the estimates are not exact, they represent a notion of kinetic similarity that is useful to explore the hierarchy of metastable states in the free energy landscape.

### Density and scale persistence

**Figure 1:**
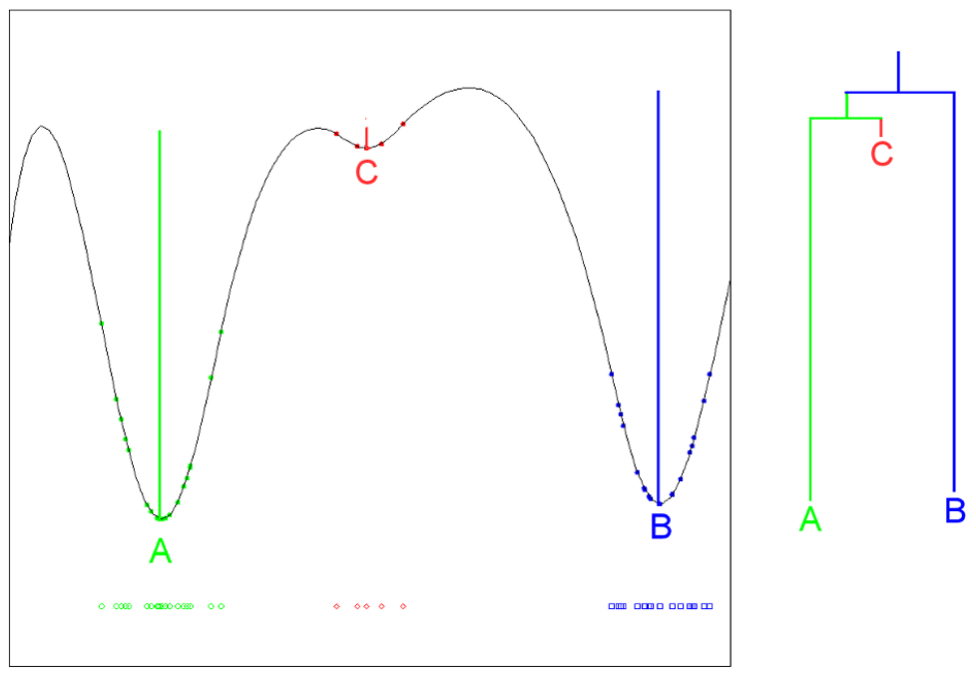
One dimensional example. A one-dimensional dataset (left lower part) and its density landscape. There are three basins in the density landscape. However, C is very shallow compared to A and B, so it might be reasonable to merge C with A to form two main clusters as shown in the right part.

To clarify the goals of our analysis, we will use a toy example. Figure 1 shows a set of microstates in a one-dimensional space, and a kernel density estimate of the free energy landscape. The plot contains information about both the relative depths of the microstates in the landscape, and how close they are to each other. It is clear that there are three minima, with a natural hierarchical structure shown to the right of the plot. State C is more shallow than the other two and is closer to A than to B. As a data exploration tool, our method would help the investigator identify the three minima and visualize this hierarchy without too much tuning. Of course, this task is more challenging when the distance is embedded in a high-dimensional space.

**Figure 2:**
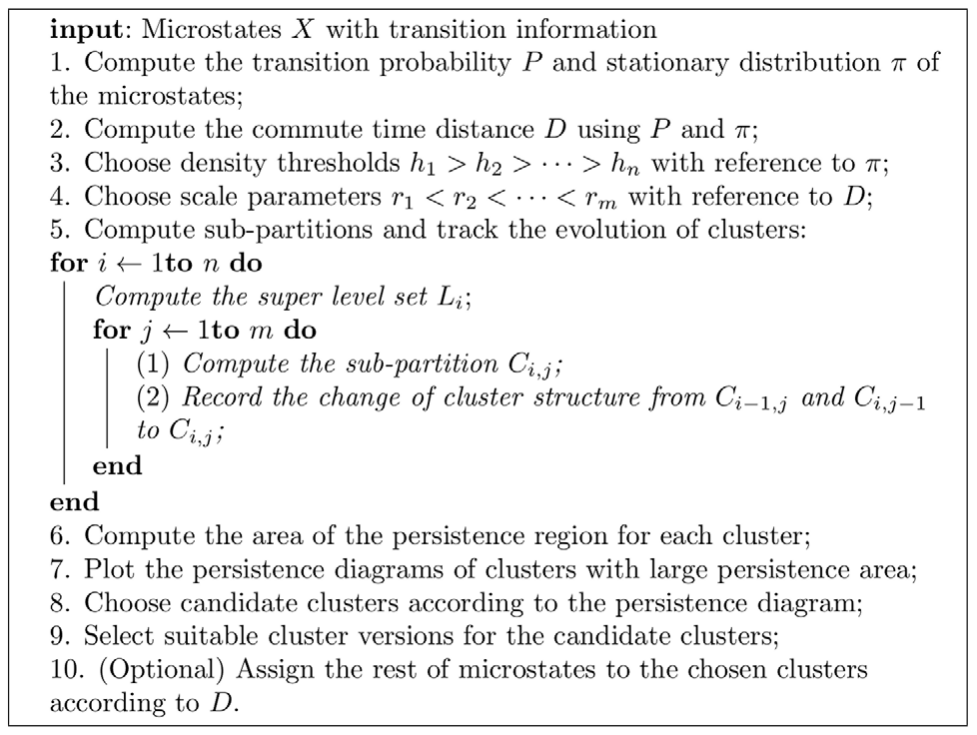
Summary of The Multi-Persistent Cluster Analysis.

The whole procedure of the MPC analysis is summarized in Figure 2, and it is explained as follows. As a proxy for the free energy of a microstate 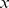, we will use the negative logarithm of the stationary probability of 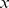 in the microstate Markov model discussed above, or 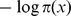. As a result, a microstate of lower free energy is a microstates with high stationary probability. In order to identify the major minima or metastable states in the system, we will evaluate the persistence of different clusters in our neighborhood clustering algorithm as we vary (i) the scale parameter, and (ii) a threshold in free energy for the microstates included in the clustering or *density parameter*. We define a sequence of *super level sets* 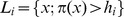 for a sequence of density parameters 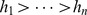. We also choose a sequence of scale parameters 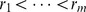. For each point 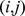 on a two-dimensional grid with 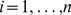 and 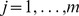, we cluster the microstates in 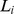 with a scale parameter 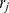.

In the neighborhood clustering algorithm, the clusters evolve in an understandable and predictable way as the parameters change. This allows us to label a cluster across all parameter settings by its microstate with the lowest free energy. As we decrease the density parameter (that is, increase the free energy threshold), we will include more microstates in the clustering procedure (if 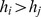, then 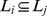). Thus, the cluster labeled by microstate 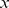 grows from the *core* of a metastable state by incorporating new microstates, until it is possibly merged by a different cluster containing a microstate 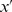 with 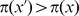. No clusterings above this point will contain the cluster labeled by 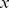. Similarly, as we increase the scale parameter, the cluster labeled by 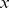 persists until it is merged by another cluster containing a microstate 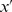 with 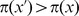. This defines a *persistence region* for each cluster in the grid of clusterings defined above. It is not difficult to prove that this region is connected.

If the commute time distance between two microstates is large, those microstates are either distant from each other on the free energy landscape or there is a big barrier between them. On the contrary, if the commute time distance between two microstates is small, they should be in the same basin of the free energy landscape. For a cluster to have long persistence in the scale parameter, the microstates in the cluster must have long commute time distance to all other microstates, and the intra-cluster commute time must be small. Therefore, a cluster that is persistent with respect to the scale parameter can be interpreted as a group of well-connected microstates which are kinetically distant from all other microstates. In other words, the system takes very long time in the cluster before leaving the cluster, which is consistent with the definition of a metastable state. Furthermore, the cluster may correspond to a single basin on the free energy landscape.

However, persistence analysis with respect to the scale parameter alone can suffer from the *chaining effect*, through which two clusters that are clearly separate are linked by states with high free energy (c.f. the Alanine Dipeptide example in the Result section). The chaining effect can be excluded if those two clusters stay separate for a large range of density parameters. Therefore, density thresholding is essential to understanding the effect of rare states or noise in the clustering. Furthermore, if a cluster has long density persistence, it must contain a microstate with very low free energy. Therefore, the cluster corresponds to a deep basin of the free energy landscape.

Because the notion of *long* persistence is relative, users should run the MPC analysis over a broad range of parameters. Fortunately, there are bounds for both the scale and density parameters. First, 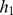 and 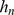 should be chosen between 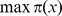 and 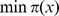. If there exists 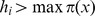, then 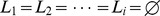. If there exists 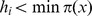, then 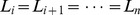 and they all contain the whole set of microstates. In both cases, we do not get more information on those level sets. Second, 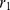 should be larger than the shortest pairwise commute time distance between the microstates; otherwise, all clusters are just singletons. On the contrary, there exists a number 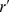 such that if the scale parameter is large than 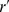 there is only one cluster in the result. Clearly, 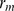 should be less than 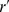.

### Choosing persistent clusters

In contrast to most clustering algorithms where the user chooses the number of clusters, the MPC analysis allows the user to choose candidate clusters directly, according to the area and shape of their persistence regions. As discussed in the previous subsection, the persistence of a cluster reflects how long the molecule system will stay in the cluster before leaving it. As a result, to construct a valid set of metastable states, users should choose the clusters with scale persistence. However, the free energy landscape is usually rugged and the MD simulation can be noisy. Therefore, it makes sense to focus on clusters with long persistence in density, which corresponds to deep basins of the free energy landscape.

Since our goal is to discover metastable states, if a cluster does not have significant persistence in the scale parameter (that is, it is not kinetically disconnected to other states) we would not choose it even though it has long persistence in the density parameter. The case of a cluster with only long persistence in the scale but not in the density parameter requires more examination. Basically, the reason for a cluster to be kinetically disconnected to other states regarding to the commute time distance is that there very few transitions between the microstates of the cluster and all other microstates in the MD simulation. It can result from a true metastable state or just because the simulation is not detailed enough to observe those transitions. In the first case, it is a small but metastable state, which could correspond to interesting intermediate states. In the latter case, the persistence will disappear when more simulation around the state is performed. This is in fact an advantage of the MPC analysis, because it does not recklessly ignore those clusters or merge them with other clusters but leaves hints about where to investigate more. This is also an important feature of a good data exploration tool.

In summary, there are three main principles in choosing clusters. First, clusters with high persistence in both dimensions are usually important. Second, if a cluster has high persistence in the scale dimension but low persistence in the density dimension, we should check whether it is merged quickly when we add more microstates or whether it simply appears late in the density dimension. In the later case, it could be a rare but kinetically distinct state. Third, we should only choose clusters with a significant number of conformations; otherwise, they might be outliers even if they show high persistence (see the Discussion section). In the following, we illustrate those principles for the alanine dipeptide dataset described in more detail in the next section.

**Figure 3:**
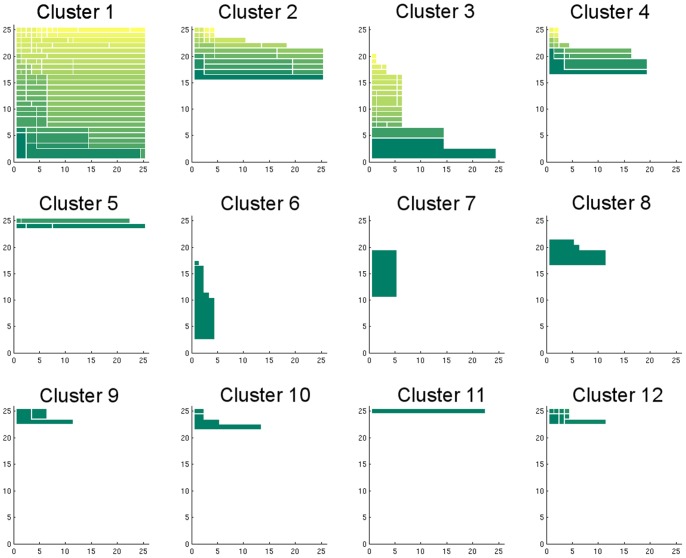
Top 12 cluster persistence diagrams (Alanine Dipeptide). The colored area in the diagram is where the cluster stays not merged by other clusters under different configurations. This figure shows the top 12 diagrams in the decreasing order of their area. A cluster starting from deeper basin of the landscape (thus is possibly a more reliable cluster) should have larger colored area. The persistence region is a single connected region; the gaps in the diagrams are just used to show the cluster merges some other cluster as the configuration changes. The color of each piece indicates the cluster size, and larger sizes are represented using color closer to yellow. The 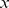-axis is the index of (increasing) scale parameters and a smaller index indicates a smaller distance threshold. The 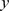-axis is the index of density thresholds and a smaller index indicates a higher (more strict) density threshold. Therefore, we can imagine more microstates are added for clustering as the level set index increases.

Figure 3 shows a collection of persistence diagrams for different clusters. The colored area in the diagram is the persistence region of that cluster, which shows under what combination of density thresholds and scale parameters the cluster is not merged by other clusters. Therefore, from a diagram one can obtain information including the energy or density level at which the miscrostate, and the corresponding cluster, appear and at which scale it get merged by other clusters. The small gaps are used to indicate steps where the cluster merges other clusters. For example, Cluster 2 in Figure 3 appears at density level 16, and it does not merge or gets merged by other clusters in the range 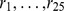 of the scale parameter at this density level. At density level 22, it merges some clusters when we increase the scale parameter from 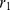 to 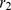, from 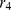 to 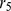, from 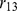 to 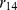, and it is merged by other clusters for scale parameters larger than 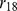.

A reliable cluster structure should have some persistence as we vary the scale and density parameters. It would be naive, however, to choose candidate clusters according to the area of their persistence region alone. The best candidate clusters are persistent in both the density and scale parameter dimensions (for instance, Clusters 1 and 3 in Figure 3). Nevertheless, we should also take into account that persistence in the density dimension is limited by how early the cluster appears. For example, Clusters 2 and 4 are good candidates as well because they are persistent under changes of the scale parameter and also in the density dimension *after they appeared*. There will always be a cluster whose persistence region covers the whole diagram (Cluster 1 in Figure 3). It is the cluster labeled by the microstate with the lowest free energy; that is, 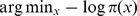.

Clusters 5 and 11 are more ambiguous. Their long persistence in the scale direction indicates they are far away from all other clusters. However, they appear only at the top of the diagram, which means they are very small compared to other ones. They might be outliers or valid but small clusters, and the decision should be made by further examination on the clusters. In fact, we will later show they are meaningful clusters, but this may not always be the case.

**Figure 4:**
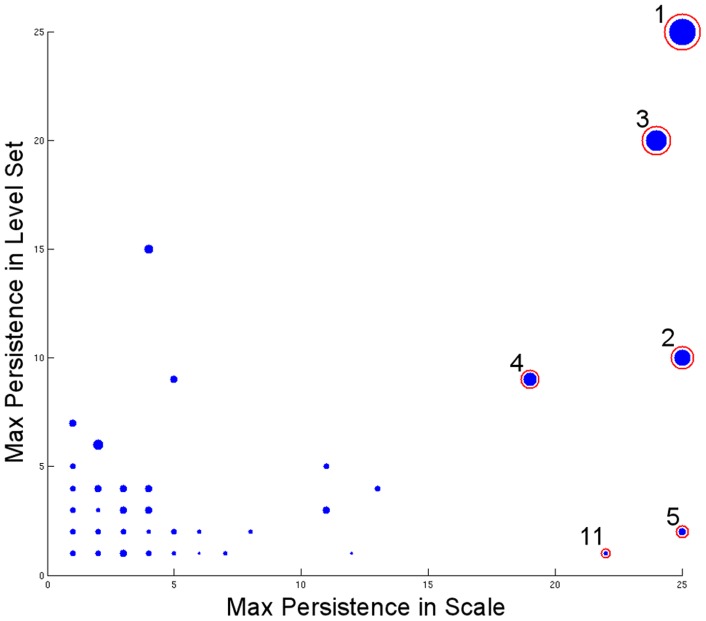
Overview of all persistence diagrams (Alanine Dipeptide). A dot is a persistence diagram. The coordinate of each diagram is assigned by its maximum persistence in the scale and level set dimensions. The 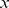-axis is the maximum length of persistence in the scale dimension, and 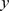-axis is maximum length of persistence in the density dimension. For example, cluster 3 has maximum scale persistence 24 (at the level sets 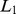 and 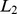) and maximum density persistence 20 (at the scale 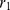). The area of a dot reflects its maximum cluster size (number of conformations in the cluster).

Recall that the number of diagrams equals the number of microstates, so it is helpful to summarize this information into the plot shown in Figure 4. Each cluster is assigned a coordinate according to the maximum length of the persistence region in the scale and density dimensions. The size of each point is proportional to the maximum number of microstates in the cluster. This visualization allows us to single out the candidate clusters 1, 3, 2, 4, 5, and 11 more easily.

### Constructing a partition of microstates

To construct a Markov model of metastable states, it is important to have a partition of microstates. In addition, a partition is convenient to visualize conformations from each metastable state. To form a partition, we must choose a version of each candidate cluster under a specific pair of scale and density parameters. The chosen version of a cluster should be as large as possible but it should not contain elements of other candidate clusters. Therefore, we will start from the candidate cluster labeled by the microstate of highest free energy and continue in order of descending free energy. For example, if we choose Clusters 1–5 and 11 in Figure 3, we will select cluster versions in the order 11, 5, 4, 3, 2, 1. The final clusters are just the core of each metastable state and their union may not contain all microstates. If a full partition of the microstates is preferred, one can assign the rest of the microstates to the chosen clusters by minimizing their commute time distance to those clusters. Finally, since the birth time and merge order of these clusters are known, one can deduce their hierarchical structure accordingly.

## Results

In this section, we apply the multiple-persistent clustering analysis explained in the previous section to MD simulations of three molecules: alanine dipeptide, Villin headpiece and FiP35 WW domain. The results for the alanine dipeptide dataset can be verified directly by visualization of the free energy landscape, which makes it an ideal model system to compare our results to the ground truth. The other two datasets are investigated in [20], where the authors checked their Markov models by comparing to published experimental results. We followed same protocol to build microstate models and test whether the cluster persistence analysis reveals the metastable states described therein. The work in [20] built MSMs with hints of the number of metastable states from the relaxation spectrum analysis, and argued both the Villin headpiece and FiP 35 WW domain have more than one native state. The aim of our analysis here is not to predict experiments but to explore and visualize the states in a microstate MSM and their kinetic/energetic relationships. Our analysis shows the multiple native states found and validated in [20] can be easily and clearly detected using our method. As a result, even if the microstate MSM is not perfect, the estimates of free energy and commute time distance derived from it is good enough for our analysis to serve as a useful exploratory tool.

### Alanine Dipeptide

**Figure 5:**
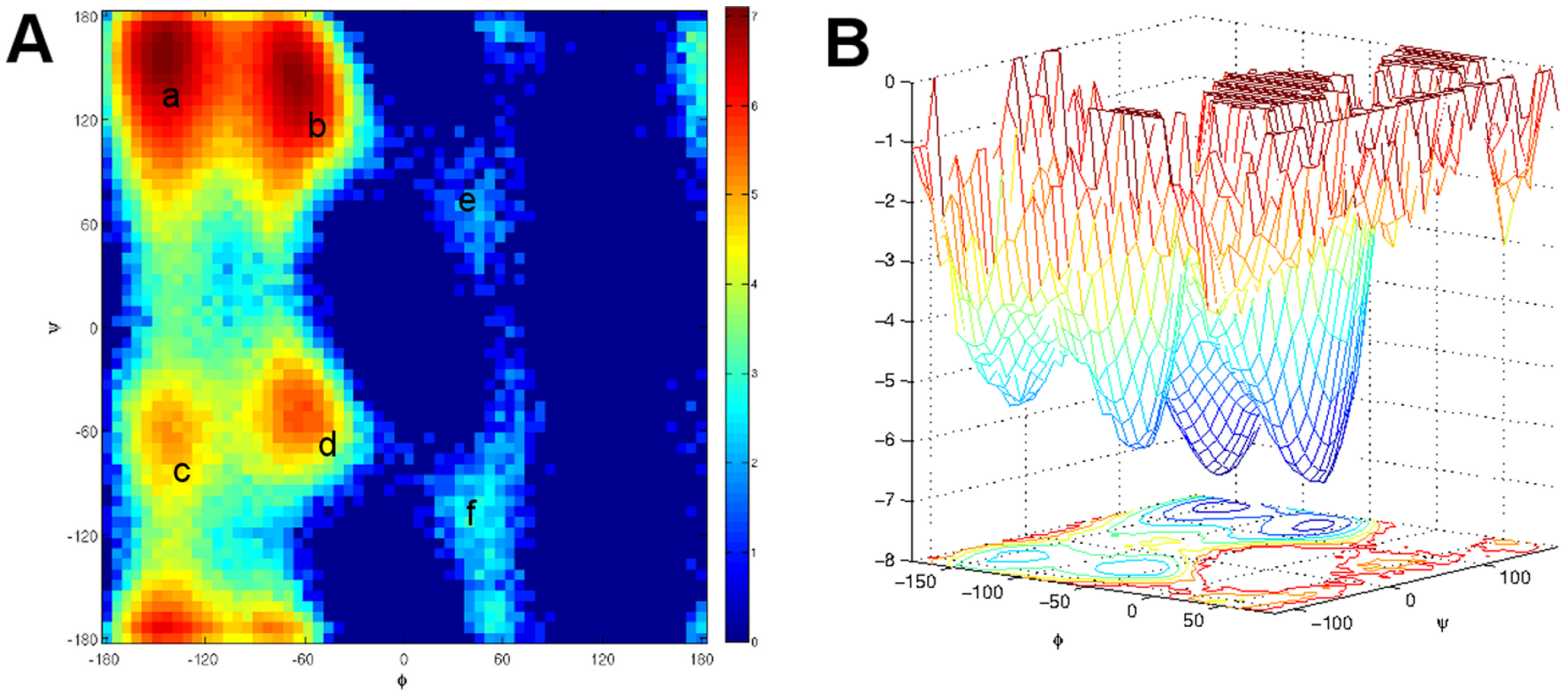
Free energy landscape (Alanine Dipeptide). (A) Density of the data on the 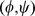 plane. There are six basins (a to f) in the density landscape. (B) Free energy landscape over the 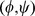 plane. The torsions angles take values in module 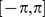; thus, the top and bottom parts of the plot are connected, so does the left and right parts.

This demonstration of the MPC analysis is done on MD simulations of the terminally-blocked alanine dipeptide in explicit solvent (sequence Ace-Ala-Nme) [14]. The alanine dipeptide system only has two slow degrees of freedom, the 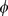 and 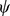 dihedral angles, so we can directly visualize the free energy landscape and the metastable states (see Figure 5). From the visualization, it is usually thought that there are six metastable states and their mapping to the 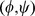 configuration space are labeled a to f in Figure 5(A). States e and f are quite small compared with the other states. Furthermore, the free energy barrier between states a and b is very low, even lower than the cores of other states. As a result, we expect that at the density level where we can see all the states, a and b are merged.

The alanine dipeptide dataset consists of 975 trajectories and each trajectory is 20 ps in length with conformations stored every 0.1 ps (200 snapshots in each trajectory). See [14] for the simulation details. The 195,000 conformations were first clustered into 1,000 microstates using the 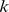-center algorithm implementation in MSMBuilder [10], [21]. Then a microstate Markov model is built with 1 ps lag time, and the MPC analysis was applied on a grid of 25 values for each, the scale and the density parameters. Our main goal is to verify that the analysis reveals the six metastable states and their relationship.

**Figure 6:**
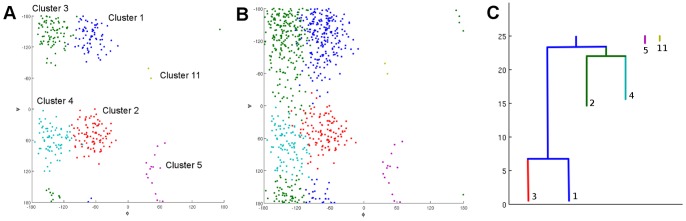
Mapping of the six chosen persistent clusters and their hierarchical relationship. (A) The projection of the cores of six chosen persistent clusters to the 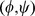 space. Each dot is the center of a microstate. (B) The full partition of the microstates into six metastable clusters. (C) The relative depth of the basins of the six selected clusters. The height corresponds to the super level set indices.

We have discussed the persistence analysis result in the previous section. Here we briefly summarize the observations in the context of previous studies on this molecule. First, recall that the cluster that covers the whole diagram (Cluster 1) is the one starting from the deepest basin and merging all other clusters in the end. In addition, there are three other clusters (Cluster 2–4) covering significant regions of the diagrams, so we can confidently establish there are at least four clusters in the data. After mapping them to the 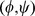 space, they correspond to the metastable states (a)–(d). The first density level where they appear also reflects the depth of corresponding basins in the free energy landscape. Then, among the other eight clusters, we see there are two clusters that do not appear until a high free energy threshold but are nonetheless persistent in the scale direction (Clusters 5 and 11). That suggests they are shallow basins but they are distant from the four main clusters; that is, they are distinct, small clusters. Indeed, if we map those two clusters, we find they correspond to metastable states (e) and (f). The corresponding clusters on the 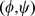 plane are shown in Figure 6(A).

The six clusters we selected do not contain all microstates. We can assign other microstates to one of the selected clusters by commute time distance as a post-processing step to obtain a full partition of the microstates. This step may not be necessary if only the core of each metastable state is needed. The full partition of microstates is shown in Figure 6(B). Comparing Figures 4(A) and 5(B), one can see they indeed reflect the manually partitioned metastable states very well. Moreover, Figure 6(C) shows the relative depth of the basins of corresponding selected clusters. The merge of two branches reflects at which free energy level the two states merge, which also gives information about the barrier height between those two states. For example, we can see from the figure that Cluster 1 and Cluster 3 have merged before other clusters appear. In addition, we can see at the last level set, Cluster 1–4 have merged.

Note these results were computed using the solute Cartesian coordinates and transition information between microstates. The dihedral angles are not explicitly used in the clustering algorithm; they are only used in the final visualization to compare the results to the ground truth clusters.

### Villin Headpiece

This dataset was obtained by sampling conformations every 10 ns from a simulation of HP35-NLE-NLE at 360 K published by Shaw et al. [3]. Beauchamp et al. [20] built a model of 900 microstates by clustering the 12,559 conformations. By a relaxation spectra analysis, the microstates are then clustered into three macrostates, which are labeled as native, near-native, and unfolded states with respective equilibrium populations 18%, 6% and 77%. According to their analysis, the conformations assigned to the native state have structures highly similar to the crystallographic model (PDB:2f4k). Furthermore, the conformations in the near-native state resemble the NMR structure (PDB:1vii, 2ppz) of the wild-type sequence, which shows partial unraveling of the C terminal helix. They further compared the results with experimental data and found consistency.

**Figure 7:**
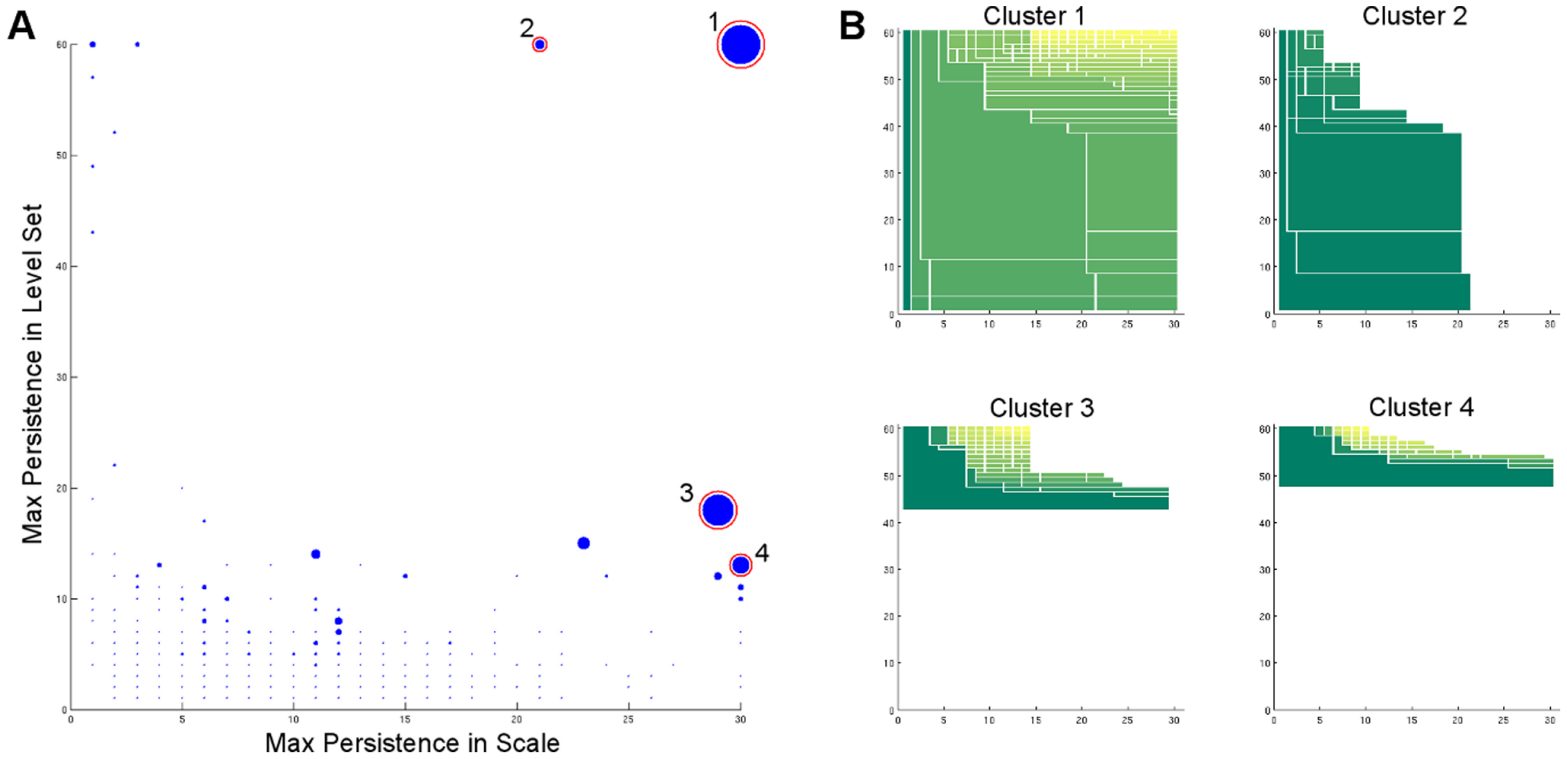
Persistence analysis of Villin Headpiece. (A) The overview of the persistence diagrams in the two dimensional persistence analysis of HP35. (B) The persistence diagrams of the 4 chosen clusters (the circled ones). The analysis is performed using 30 scale parameters and 60 level sets.

**Figure 8:**
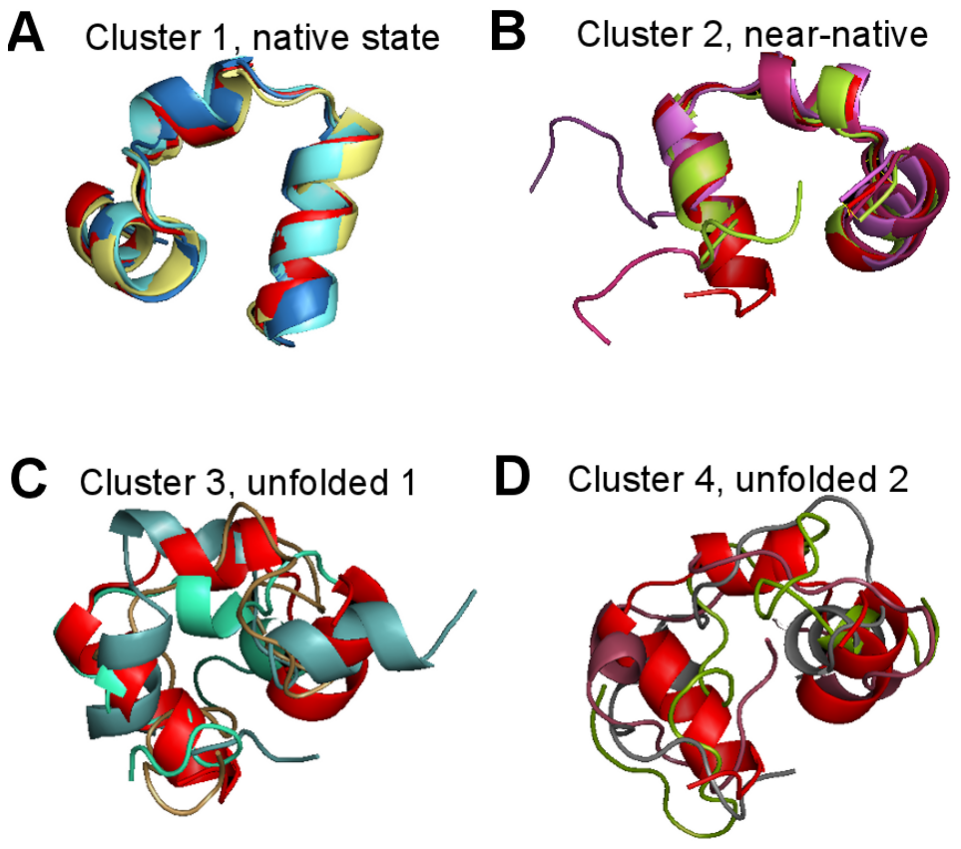
Structures of Villin Headpiece in the clusters. (A) The alignment of three randomly sampled conformations from Cluster 1 to the native structure (shown in red). (B) The alignment of three randomly sampled conformations from Cluster 2 to the near native structure (shown in red). (C) Three randomly sampled conformations from Cluster 3. (D) three randomly sampled conformations from Cluster 4.

We performed the two-dimensional cluster persistence analysis on the same microstates obtained from the authors of [20]. It is clear in Figure 7 there are only two clusters with long persistence in both the scale and density parameters (labeled Cluster 1 and Cluster 2). After comparison with the model in [20], we found Cluster 1 corresponds to the native state, Cluster 2 corresponds to the near-native state, and Clusters 3 and 4 correspond to the unfolded state. In Figure 8, we randomly sampled three conformations from each of the four selected clusters and aligned them with the native structure (PDB:2f4k, shown in red in the figures). The conformations from Cluster 1 strongly resemble the native structure and conformations from Cluster 2 resemble the near-native state identified in [20].

### FiP35 WW Domain

This dataset was obtained by sampling conformations every 50 ns from the simulation of FiP35 WW domain at 395 K published by Shaw et al. [3]. Beauchamp et al. apply the same clustering procedure to generate a microstate model of 290 states from 4,000 conformations, and a three-state model of the protein [20]. The authors reported that two of those macrostates show good agreement with NMR structures (holo PDB:1i8g, apo PDB:1i6c) of the related Pin WW domain, and the third one is labeled as the unfolded state.

**Figure 9:**
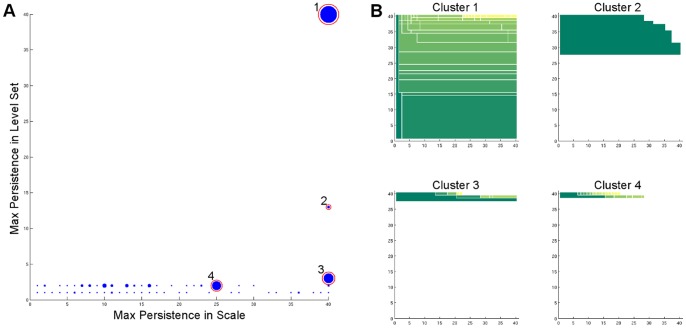
Persistence analysis of FiP35 WW Domain. (A) The overview of the persistence diagrams in the two dimensional persistence analysis of FiP35. (B) The persistence diagrams of the 4 chosen clusters (the circled ones). The analysis is performed using 40 scale parameters and 40 level sets.

**Figure 10:**
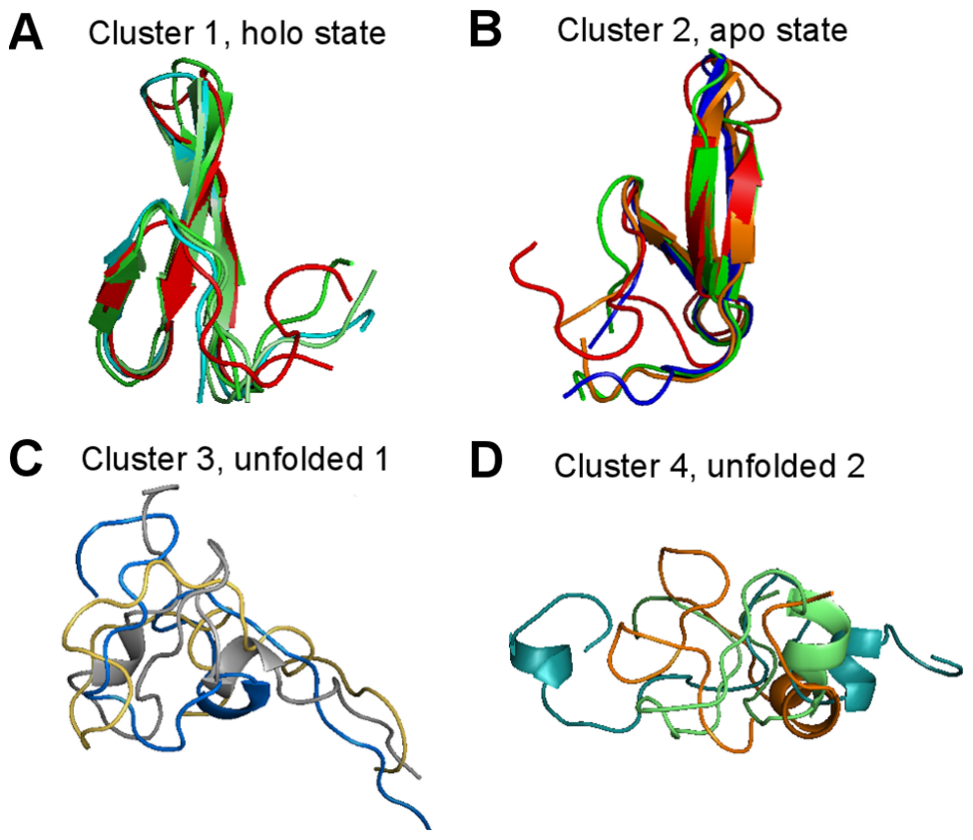
Structures of FiP35 WW Domain in the clusters. (A) The alignment of three randomly sampled conformations from Cluster 1 to the holo structure (PDB:1i8g) shown in red. (B) The alignment of three randomly sampled conformations from Cluster 2 to the apo structure (PDB:1i6c) shown in red. (C) Three randomly sampled conformations from Cluster 3. (D) three randomly sampled conformations from Cluster 4.

From the persistence diagram in Figure 9, we choose four clusters. After comparing with the PDB structures, we found that Cluster 1 and Cluster 2 show strong similarity to the holo and apo states, respectively. In addition, Clusters 3 and 4 correspond to the unfolded states of the three-state model in [20]. Again, in Figure 10 we show three randomly sampled structures from each of the four selected clusters, and the red structures in Figure 10(A) and Figure 10(B) are the holo (PDB:1i8g) and apo (PDB:1i6c) structures, respectively. Note although from the visualization it seems that there is some structures in Cluster 3 and 4, it might be misleading because they are in fact very small clusters (c.f. the persistence diagrams and the Discussion section). Furthermore, the original dataset only contains 4,000 conformations; to obtain a more reliable hint about the cluster structure beyond the holo and apo states, more data are needed.

## Discussion

Clustering stability with respect to perturbations of data is a commonly used criterion for model selection and its theoretical foundation has been studied in several papers including [22]–[24]. A related concept in the biomolecular data clustering context can be found in [25]. Furthermore, relevant stability theorems about the scale persistence in our method are described in [26]. Investigating clusters at different density levels is known as *cluster tree analysis* in statistics. Recently, several algorithms for merging short branches of a cluster tree to find statistically significant clusters have been published and much progress has been made in their theoretical justification [27]–[31]. However, most existing methods either require users to provide a hard threshold to decide when to merge or ignore the distance structure at all. If a small cluster is far away from all other big clusters, it should be regarded as a cluster without sufficient samples or just an outlier. Incorrect merging may bias the structure of the cluster that it is merged to. Therefore, our cluster persistence analysis gives a better solution to the problem by providing a multiresolution view of the clusters, which allows the user to make an informed decision.

Clusters whose microstates have low stationary probability, and thus have low persistence in the density direction, should be chosen with care even they have significant persistence in the scale dimension. Although they may correspond to meaningful rare states, as in the alanine dipeptide example, the persistence in the scale dimension could also be an artifact of noise in the distance function. In fact, microstates with low stationary probability have fewer observed transitions, which results in large commute times to other microstates and misleading persistence in the scale direction. This could be the reason why there are multiple unfolded states in our HP35 and FiP35 results compared with three-state models built using PCCA related methods [20].

The main ideas behind two-dimensional persistence analysis can be combined with other clustering methods. For example, we could use the selected cores as seeds and assign other microstates in a semi-supervised fashion. Another possibility is to build a simple model using PCCA or its variants and then apply the analysis on the resulting metastable states to discover intermediate states. Furthermore, the persistence analysis is not restricted to microstate clustering. One could apply the analysis to cluster all the conformations observed in a simulation using any distance function which captures structural or kinetic similarities between them.

## Conclusions

Two-dimentional cluster persistence analysis is a useful tool for exploratory analysis of MD simulations. In the proposed pipeline, the number of clusters is not determined before running the clustering algorithm, but selected by the user according to the analysis of cluster persistence. The main native states of the system can then be chosen with confidence. For the clusters on the borderline the user can make a decision based on their structural characteristics. The clusters chosen according to their persistence are the clusters that are least sensitive to perturbation of the data or distance function. Furthermore, the multi-resolution analysis gives users the information of the relative potential of the clusters and thus their hierarchical relationship. In the Alanine Dipeptide dataset, the method discovers six clusters that are strongly consistent with the manual decomposition of the free energy landscape. Furthermore, the results of applying the method to the HP35 and FiP35 simulation data also identifies native or near-native states which are consistent with published results.
